# Postoperative quadriceps weakness and male sex are risk factors for patellofemoral articular cartilage lesions after anatomical anterior cruciate ligament reconstruction

**DOI:** 10.1007/s00167-023-07633-3

**Published:** 2023-10-26

**Authors:** Ryo Murakami, Shuji Taketomi, Ryota Yamagami, Kenichi Kono, Kohei Kawaguchi, Tomofumi Kage, Takahiro Arakawa, Hiroshi Inui, Sakae Tanaka

**Affiliations:** Department of Orthopaedic Surgery, Faculty of Medicine, The University of Tokyo, 7-3-1 Hongo, Bunkyo-Ku, Tokyo, 113-8655 Japan

**Keywords:** ACL, PF, Patellofemoral, Cartilage, Cartilage lesions

## Abstract

**Purpose:**

Patellofemoral (PF) compartment cartilage lesions are a frequent problem after anterior cruciate ligament (ACL) reconstruction. This study aimed to determine the factors that influence PF cartilage lesions after anatomical ACL reconstruction.

**Methods:**

This study enrolled a total of 114 patients who did not manifest PF compartment cartilage lesions during anatomical ACL reconstruction and underwent second-look arthroscopy 18 months postoperatively. Arthroscopy using the International Cartilage Repair Society (ICRS) classification was used to assess cartilage lesions. The correlation between surgical findings, radiographic factors, and clinical factors and change of ICRS grade was analysed. Multivariate regression analysis was conducted to reveal the independent risk factors for PF cartilage lesions among patients’ demographic data and parameters that correlated with the change of ICRS grade in the correlation analyses.

**Results:**

ICRS grade changes in PF cartilage were significantly correlated with age, sex, quadriceps strength at 1 year postoperatively, hamstrings strength at pre- and 1 year postoperatively, and single leg hop test at 1 year postoperatively. However, no significant correlation was found between the time between injury and surgery, posterior tibial slope angle, pre- and postoperative Tegner activity scale, graft type, initial graft tension, meniscus injury, meniscus injury treatment, pre- and postoperative range of motion, anteroposterior laxity and preoperative quadriceps strength, and the change in ICRS grade. Multivariate regression analysis revealed male (*P* = 0.019) and quadriceps strength weakness at 1 year postoperatively (*P* = 0.009) as independent risk factors for PF cartilage lesions.

**Conclusions:**

Quadriceps strength weakness 1 year after ACL reconstruction and males were correlated with a new PF cartilage lesion after anatomical ACL reconstruction, with no significant correlation between bone-patellar tendon-bone autograft, initial graft tension, or extension deficit and new PF cartilage lesion. Rehabilitation that focuses on quadriceps strength after ACL reconstruction is recommended to prevent new PF cartilage lesions.

**Level of evidence:**

Level IV.

## Introduction

Patellofemoral (PF) cartilage lesions after anterior cruciate ligament (ACL) reconstruction are sometimes detected at second-look arthroscopy and cause symptoms that negatively affect sports activities, and especially PF cartilage lesions may be more than tibiofemoral (TF) cartilage [4, 25]. Many factors, such as patient characteristics, and surgical factors have been reported in PF cartilage lesions after ACL reconstruction [8, 12, 23, 31]. Extension deficit and quadriceps weakness caused by inadequate rehabilitation alter the PF joint biomechanics and cause PF cartilage lesion progression [4]. Similarly, initial graft tension has recently been identified as one of these factors because initial graft tension affects the PF joint biomechanics [6, 9]. Bone-patellar tendon-bone (BPTB) autograft is often reported as one of these factors because of extensor mechanism weakness caused by BPTB harvest [9]. However, this systematic review encompassed numerous articles about nonanatomical ACL reconstruction, which is one of these factors and produces abnormal knee kinematics and leads to cartilage lesions [2, 29]. A few studies reported on PF cartilage lesions after anatomical ACL reconstruction, and the cause of PF cartilage lesions after anatomical ACL reconstruction is unclear.

Moreover, the condition of the cartilage at the primary ACL reconstruction is important for evaluating PF cartilage lesions. The presence of cartilage lesions during ACL reconstruction is a significant factor in cartilage lesion progression, irrespective of surgery [1, 7, 32]. Therefore, pre-existing cartilage lesion progression should be distinguished from the occurrence of new cartilage lesions during the postoperative period. However, most studies on PF articular cartilage lesions after ACL reconstruction included patients with some PF or TF compartment cartilage lesions.

This study aimed to determine the factors influencing the PF cartilage lesions after anatomical ACL reconstruction with arthroscopic evaluation in patients who have normal PF cartilage at primary surgery. The hypothesis of this study was that there would be an increased risk of PF cartilage lesions after anatomical ACL reconstruction due to extension deficit, quadriceps strength weakness, and higher initial graft tension, but BPTB autograft would not increase the risk. The results of this study contribute to preventing new PF cartilage lesions by identifying risk factors and ensuring appropriate interventions.

## Materials and methods

The institutional review board (approval number 2674, the University of Tokyo, Bunkyo-ku, Tokyo, Japan) approved this study. A total of 254 consecutive patients underwent primary ACL reconstruction using a BPTB autograft or a hamstrings tendon (HT) autograft and second-look arthroscopic assessment and hardware removal at our institution from February 2010 to August 2022. The exclusion criteria were (1) second-look arthroscopy within 18 months after ACL reconstruction, (2) International Cartilage Repair Society (ICRS) grade [16] of ≥ 1° in PF compartment cartilage lesions or ≥ 2° in TF compartment cartilage lesions at a primary ACL reconstruction, (3) the presence of posterior sagging and abnormal varus/valgus instability, (4) history of patellar dislocation or patellar maltracking, and/or (5) history of ligament injury of the contralateral knee, accounting 51, 80, 1, 0, and 8 patients, respectively. Hence, 114 patients were finally included in this study. Patients included 74 males and 40 females, with the mean age being 27.1 years (range: 15–55 years). The median preinjury Tegner activity scale was 7 (range: 3–10). The median time of second-look arthroscopy was 25 months (range: 18–64 months). No trauma was noted around the bilateral knee joints between the ACL reconstruction and second-look arthroscopy. ACL reconstructions using BPTB autografts were performed in 89 patients and ACL reconstructions using HT autografts in 25 patients. All procedures were performed by several knee surgeons using the same surgical technique. An experienced senior surgeon (T.S.) participated in all the procedures either as the chief surgeon or first assistant.

### Clinical evaluation

Covariates included age, sex, body mass index (BMI), Tegner activity scale, time between injury and surgery (recorded in months), time of second-look arthroscopy (recorded in months), graft material, initial graft tension, medial meniscal status, lateral meniscal status, treatment for each meniscus, preoperative and postoperative side-to-side differences (SSD) in the range of motion (ROM) and anterior tibial translation using an arthrometer, preoperative and postoperative limb symmetry index (LSI) in quadriceps and hamstrings strength, and postoperative LSI in single leg hop test. ROM and anteroposterior laxity were expressed as SSD (operated—contralateral limb). A goniometer was used to evaluate ROM for knee extension and flexion angle preoperatively, 1 year postoperatively, and at second-look arthroscopy. SSD was calculated for knee extension and flexion ROM and values were classified as < 5° SSD or ≥ 5° SSD. Anteroposterior laxity was reported as the SSD between the two limbs and measured with Kneelax3 (Monitored Rehab Systems, Haarlem, the Netherlands) preoperatively and 6 months, 1 year, and 2 years postoperatively. The SSD in the anteroposterior tibial displacement between the injured knee and the contralateral knee was measured with an anterior force of 134 N applied to the proximal tibia at 20° of knee flexion and calculated in millimetres. Muscle strength and single leg hop test were expressed as an LSI. LSI was calculated by dividing operated limb data by contralateral limb data and then multiplying by 100. Quadriceps and hamstrings strength were assessed as peak knee extension and flexion torque at 60°/s with the Cybex (Lumex, Ronkonkoma, New York, USA) preoperatively and 6 months and 1 year postoperatively. ROM at testing was set at 0°–90° of knee flexion at a speed of 60°/s. Patients performed five repetitions of knee extension and flexion for each leg after performing a warm-up for 5 min, and the maximum peak extension torque of five repetitions at 60°/s was recorded. The single leg hop test described by Noyes et al. [19] was bilaterally measured 1 year postoperatively. The LSI was calculated from the average of three successful measurements and utilised for data analysis.

### Radiographic evaluation

**Fig. 1:**
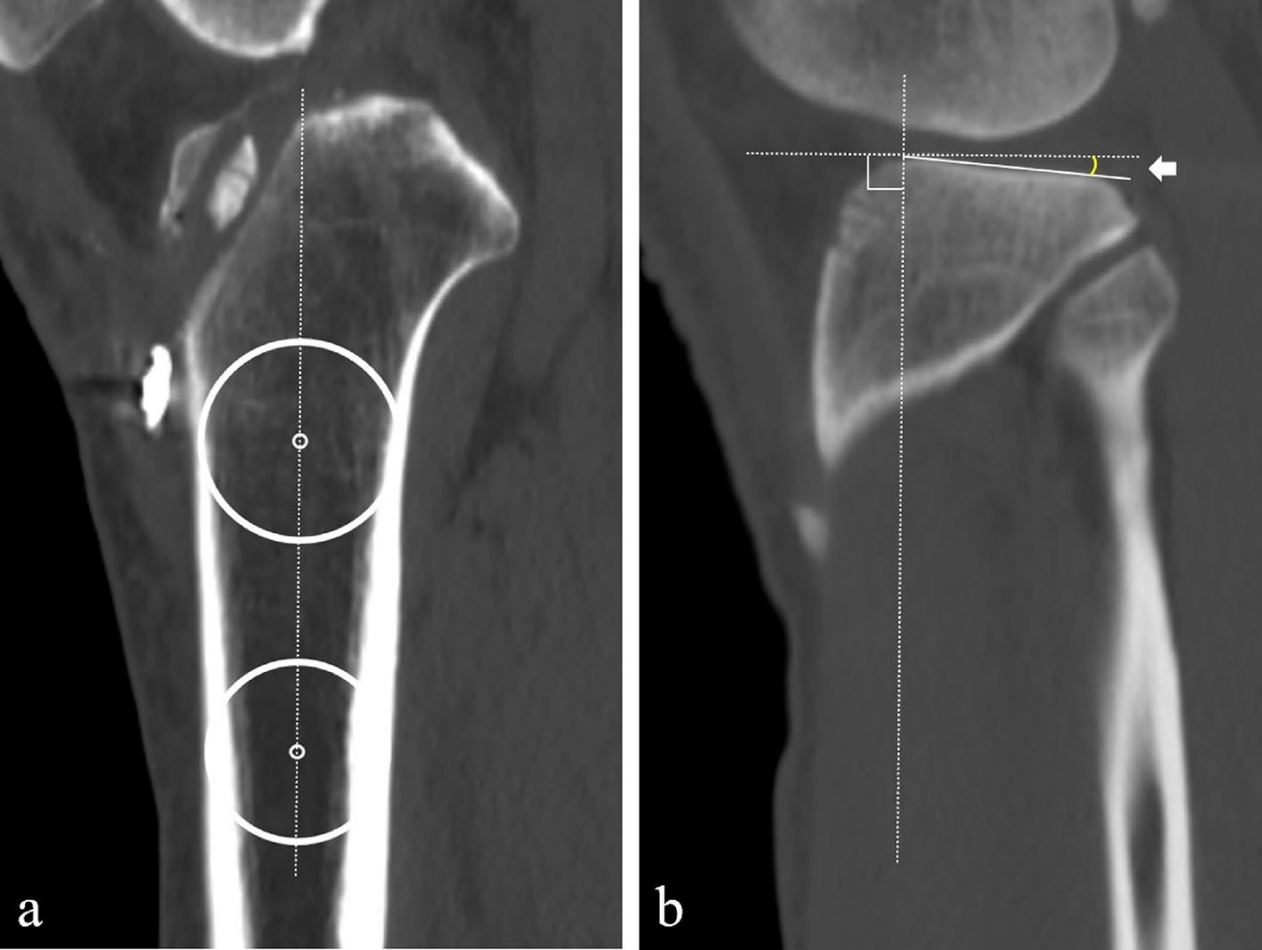
PTS measurement on CT images. **a** The longitudinal tibial axis was determined by drawing two circles. These two circles were tangent to the anterior and posterior cortical border at 5 cm and 10 cm points distal to the knee joint. A line connecting the centres of the two circles defined the longitudinal tibial axis. **b** The angle between the axis perpendicular to the longitudinal axis and the line connecting the most proximal anterior and posterior subchondral bone points of the medial and lateral tibiofemoral compartments determined the medial and lateral PTS. *PTS* posterior tibial slope, *CT* computed tomography

Computed tomography (CT) on the knee joints was performed for all patients with their knees extended 1 week postoperatively. Helical high-speed Aquilion PRIME, Aquilion precision, or Aquilion ONE (Toshiba Medical Systems Co., Japan) CT machines were used for CT scan. The medial and lateral posterior tibial slope (PTS) were measured using CT with a modification of the previously reported method using the ImageJ software (National Institutes of Health, Bethesda, MD) [14, 18]. The longitudinal tibial axis was assessed on the central slice on sagittal views, with the intercondylar eminence, anterior tibial cortices, and posterior tibial cortices appearing as a concave shape. The longitudinal tibial axis was determined by drawing two circles. The two circles were tangent to the anterior and posterior cortical border at 5 cm and 10 cm points distal to the knee joint. A line connecting the centres of the two circles defines the longitudinal tibial axis (Fig. 1a). A coronal view was used to select the mid-sagittal images of the medial and lateral TF compartments. The angle between the axis perpendicular to the longitudinal axis and the line connecting the most proximal anterior and posterior subchondral bone points of the medial and lateral TF compartments determined the medial and lateral PTS (Fig. 1b).

### Surgical procedure of ACL reconstruction with the BPTB graft

The ACL reconstruction with the BPTB graft has been previously described [28]. The femoral bone plug was formed into a 5 or 6 × 10 mm rectangular shape after harvesting autologous BPTB grafts in 10 mm of width from the central portion of the patellar tendon, as described by Shino et al. [26].

**Fig. 2:**
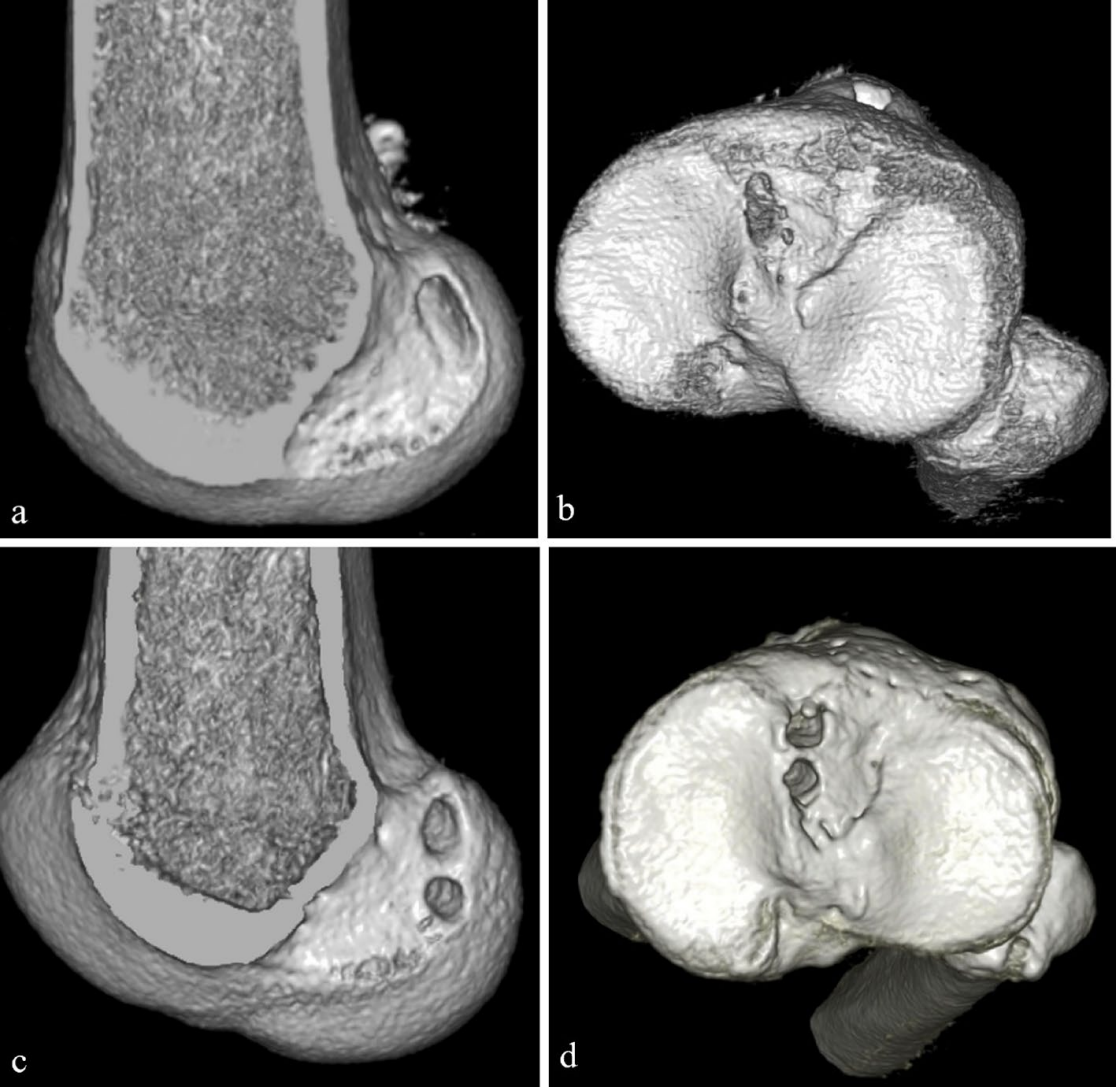
Representative tunnel positions on three-dimensional computed tomography images. **a** Femoral tunnel during ACL reconstruction with BPTB autograft. **b** Tibial tunnel with BPTB autograft. **c** Femoral tunnels with HT autograft. **d** Tibial tunnels with HT autograft. *ACL* anterior cruciate ligament, *BPTB* bone-patellar tendon-bone, *HT* hamstrings tendon

The femoral tunnel was created as far posterior and proximal as possible to the ACL femoral footprint, which is posterior to the resident ridge and anterior to the articular cartilage margin. Two guidewires were inserted in parallel through the far anteromedial (FAM) portal and drilled to the appropriate length with a 5 or 6-mm cannulated drill, respectively, and a dilator was used to connect the two bony tunnels and made rectangularly (Fig. 2a). The tibial tunnel was created as far anterior and medial to the ACL tibial footprint as possible, referring to the anterior horns of the medial and lateral meniscus, the medial intercondylar ridge, and Parsons’ knob. Two guidewires were inserted in parallel and drilled with a cannulated drill, and two tunnels were connected with a dilator to form a rectangle (Fig. 2b). Endobutton (Smith & Nephew, Andover, MA, USA) was used to secure the femoral side to make the distal end of the bone plug on the femoral side 1 mm from the inside of the joint. Until 2012, a suture post-fixation with a half-threaded 6.5-mm cancellous screw and washer (Meira Corp., Nagoya, Japan) was secured to fix the tibial side. The graft was secured at full knee extension with manual maximum pull (higher tension protocol). Since 2013, the tibial side was secured using a double-spike plate small (Smith & Nephew) and a half-threaded 5-mm cancellous screw. The graft was secured at 20° knee flexion so that the same tension is applied as if the BPTB graft was pulled with an 80 N pull at full knee extension using a ligament tensioner (Smith & Nephew). The tension at 20° knee flexion varied from patient to patient and ranged from 5 to 20 N pull (lower tension protocol).

### Surgical procedure of ACL reconstruction with the HT graft

The ACL reconstruction with the HT graft has been previously described [28]. Double grafts were looped over Endobutton CLs (Smith & Nephew) after harvesting autologous semitendinosus and gracilis grafts. The femoral and tibial tunnel orientation was the same as in ACL reconstruction with the BPTB graft. Two femoral tunnels were created from the FAM portal (Fig. 2c) and two tibial tunnels were created using the outside-in technique (Fig. 2d). Until 2012, the graft was secured at full knee extension with manual maximum pull (higher tension protocol). Since 2013, each graft was secured at 20° knee flexion so that the same tension is applied as if each bundle was pulled with a 40 N pull at full knee extension using a ligament tensioner. The tension at 20° knee flexion for each bundle varied from patient to patient and ranged from 3 to 15 N pull (lower tension protocol). The same fixation device on the tibial side was used as that in ACL reconstruction with a BPTB graft for each period.

### Postoperative rehabilitation

ROM exercises were immediately initiated postoperatively. Partial-weight-bearing gait was permitted at 2 days and full-weight-bearing gait at 1 week. A functional knee brace was used for 6 weeks postoperatively. A return to sports was permitted at an average of 8–9 months postoperatively, with running permitted at 4 months. Full-weight-bearing gait was allowed at 6 weeks for patients who underwent a concomitant repair of a meniscus radial tear. Other routine or daily activities were maintained accordingly.

### Second-look arthroscopic evaluation

**Fig. 3:**
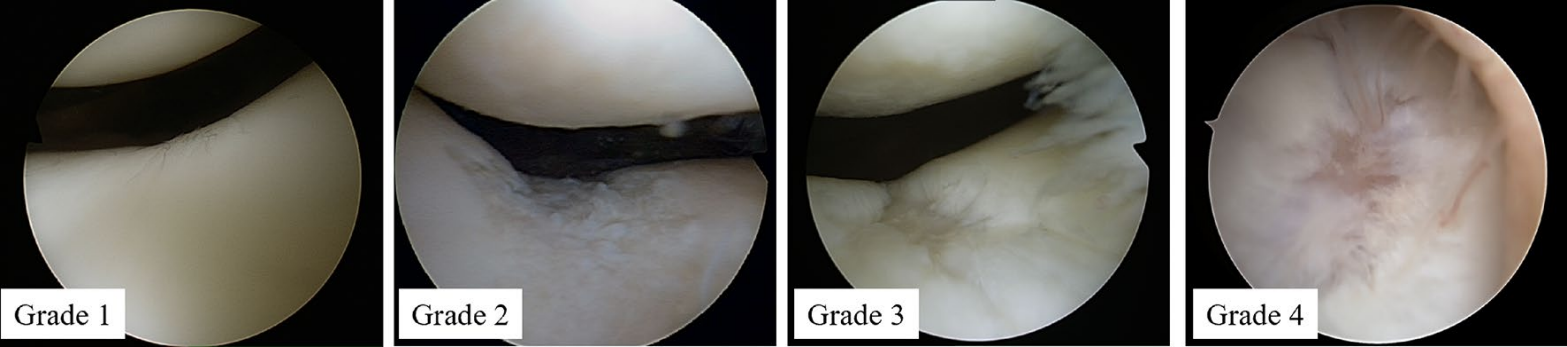
Evaluation of cartilage lesions. Cartilage was assessed by arthroscopy, and cartilage lesions were evaluated with the International Cartilage Repair Society articular cartilage injury classification (Grades 0–4). This study revealed no cases of cartilage lesions with ICRS grade of 4

Second-look arthroscopy and hardware removal were performed usually at approximately 24 months after the primary ACL reconstruction. Most patients did not complain of intra-articular symptoms. ICRS articular cartilage injury classification (Grades 0–4) was used for arthroscopic grading to evaluate cartilage degeneration, and cartilage lesion grading was assessed by the chief surgeon and first assistant (either of which always included a senior surgeon [T.S.]). The conclusion was only achieved at the agreement of both (Fig. 3). Chondral lesions in the PF component at primary ACL reconstruction and at second-look arthroscopy were compared and the change of ICRS grade was evaluated.

### Statistical analysis

Bell curve for Excel (Social Survey Research Information Co., Ltd. Tokyo, Japan) was used for statistical analysis. Statistical significance was set at *P*-values of < 0.05. Pearson’s correlation coefficients (r) were conducted to evaluate the correlation between the change in ICRS grade and outcome measures. Multivariate regression analysis was conducted to detect the factors that significantly correlated to the change of ICRS grade among age, sex, BMI, and other parameters found to correlate with the change of ICRS grade in the univariate analyses. To determine the power of this study, post hoc power analysis was performed using G*Power (Version 3.1.9.7; HHU). Given the number of predictor valuables and sample size of 114 with an effect size of 0.15 and α-level of 0.05, a power of 0.85 was obtained. This study randomly selected 10 sets of CT scans to determine the reproducibility of the PTS measurements. Interobserver and intraobserver reliabilities were assessed using intraclass correlation coefficients (ICCs). The same investigator repeated the evaluations after 3 weeks. The second investigator repeated the same evaluation protocols. The inter- and intraobserver ICCs were 0.977 and 0.979 for the medial PTS and 0.942 and 0.973 for the lateral PTS, respectively.

## Results

Table 1 shows the changes in ICRS grade between ACL reconstruction and second-look arthroscopy. The patients’ demographic data are presented, and correlation analysis among the patients’ demographic data revealed sex and age as significant correlations with the change of ICRS grade (*r* = 0.25, *P* < 0.01 and *r* = 0.20, *P* = 0.03, respectively) (Table 2). No significant correlations were found with the change of ICRS grade among the surgery factors, such as graft type, initial graft tension, meniscus tear, and treatment for meniscus. Additionally, correlation analysis among the ROM, anteroposterior laxity, muscle strength, and single leg hop test revealed that quadriceps strength LSI at 1 year postoperatively (*r* =  − 0.41, *P* < 0.01), hamstrings strength LSI preoperatively and 1 year postoperatively (*r* =  − 0.22, *P* = 0.04 and *r* =  − 0.23, *P* = 0.01 respectively), and single leg hop test LSI 1 year postoperatively (r =  − 0.29,* P* < 0.01) demonstrated a significant negative correlation with the change of ICRS grade (Table 3). Multivariate regression analysis was conducted using age, sex, BMI, quadriceps strength LSI 1 year postoperatively, hamstrings strength LSI preoperatively and 1 year postoperatively, and single leg hop test LSI 1 year postoperatively. Multivariate regression analysis demonstrated a significant difference between the change of ICRS grade and the quadriceps strength LSI 1 year postoperatively (*P* = 0.009) and sex (*P* = 0.019) (Table 4). Lower quadriceps strength LSI 1 year postoperatively and males were significantly correlated with the PF compartment cartilage lesions after anatomical ACL reconstruction.

**Table 1:** The change of ICRS grade

Change of ICRS grade	
0 (0 → 0)	74
1 (0 → 1)	27
2 (0 → 2)	9
3 (0 → 3)	4
4 (0 → 4)	0

*ICRS* International Cartilage Repair Society

^*^P value < 0.05

^**^P value < 0.01

**Table 2:** Patients’ demographic data and correlation with the change of ICRS grade

			*P* value
Sex (Male/female)		74/40	< 0.01
Age (years)		27.0 ± 10.1	0.03
BMI (kg/m^2^)		23.0 ± 3.2	n.s
Posterior tibial slope (°)	Medial	7.7 ± 2.7	n.s
	Lateral	8.2 ± 3.3	n.s
Tegner activity scale	Pre	7 (6–9)	n.s
	Post	7 (6–8)	n.s
Time between injury and surgery (months)		4 (2–7)	n.s
Time of second-look arthroscopy (months)		25 (22–30)	n.s

Data are presented as number of patients, mean ± standard deviation, or median (range)

*ICRS* International Cartilage Repair Society, *BMI* body mass index, *n.s.* not significant

**Table 3:** Correlation between surgical findings and clinical factors and the change of ICRS grade

			*P* value
Graft type (HT / BPTB)		25/89	n.s
Initial graft tension (Higher / Lower)		26/88	n.s
MM tear	+ / −	34/80	n.s
	Suture/resection/none	25/9/80	n.s
LM tear	+ / −	34/80	n.s
	Suture/resection/none	24/10/80	n.s
Extension ROM SSD ≥ 5° ( ±)	Preoperative	13/101	n.s
	1 year	3/111	n.s
	Second-look	3/111	n.s
Flexion ROM SSD ≥ 5° ( ±)	preoperative	32/82	n.s
	1 year	7/107	n.s
	Second-look	5/109	n.s
Anteroposterior laxity SSD (mm)	Preoperative	3.9 ± 2.1	n.s
	6 months	0.5 ± 1.6	n.s
	1 year	0.3 ± 1.6	n.s
	2 years	0.7 ± 1.3	n.s
Quadriceps strength LSI (60°/s) (%)	Preoperative	78.0 ± 23.2	n.s
	1 year	84.2 ± 16.2	< 0.01
Hamstrings strength LSI (60°/s) (%)	Preoperative	82.4 ± 23.0	0.04
	1 year	95.0 ± 14.6	0.01
Single leg hop test LSI 1 year postoperatively (%)		90.5 ± 10.3	< 0.01

Data are presented as number of patients or mean ± standard deviation

*ROM* range of motion, *ICRS* International Cartilage Repair Society, *HT* hamstrings tendon, *BPTB* bone-patellar tendon-bone, *MM* medial meniscus, *LM* lateral meniscus, *SSD* side-to-side differences, *LSI* limb symmetry index, *n.s.* not significant

**Table 4:** Results of the multivariate regression analysis

	Estimate	Standard Error	95% CI	*P* value
Sex (Male vs Female)	0.416	0.173	(0.072, 0.760)	0.019
Age	0.010	0.008	(− 0.005, 0.024)	n.s
BMI	− 0.013	0.026	(− 0.064, 0.038)	n.s
Quadriceps strength LSI 1 year postoperatively	− 0.016	0.005	(− 0.025, − 0.004)	0.009
Hamstrings strength LSI preoperatively	− 0.002	0.004	(− 0.009, 0.005)	n.s
Hamstrings strength LSI at 1 year postoperatively	− 0.007	0.006	(− 0.018, 0.004)	n.s
Single leg hop test LSI at 1 year postoperatively	− 0.009	0.008	(− 0.026, 0.007)	n.s

*CI* confidence interval, *BMI* body mass index, *LSI* limb symmetry index, *n.s.* not significant

## Discussion

The most significant finding of this study was the correlation between quadriceps strength weakness at 1 year after ACL reconstruction or the male sex and new PF cartilage lesion after anatomical ACL reconstruction although with no significant correlation between BPTB autograft, initial graft tension, or extension deficit and new PF cartilage lesion.

It was revealed that the male sex is a negative risk factor for PF cartilage lesions in this study, and several magnetic resonance imaging (MRI) studies have indicated that males have a risk of developing PF cartilage lesions [3, 21]. Regarding biomarkers, the matrix–metalloproteinase-3 (MMP-3) levels in females showed no significant differences between preoperative and return to activity after ACL reconstruction, while those in males significantly increase from preoperative to return to activity [23]. However, the mechanism that males affect the PF cartilage lesions is unclear and further research regarding the correlation between sex and PF cartilage lesions is required.

Several articles support that quadriceps muscle strength affects PF articular cartilage injury. Quadriceps strength weakness at the final follow after ACL reconstruction has been reported as a factor associated with PF osteoarthritis (OA) [12]. In addition, a quadriceps strength LSI of < 80% at the last follow-up was associated with patella cartilage damage progression [8, 31]. Further, the present study revealed that quadriceps strength weakness 1 year postoperatively affected the new PF compartment cartilage lesion after ACL reconstruction, consistent with the results of previous studies.

BPTB autograft is often reported as a risk factor for PF joint cartilage damage after ACL reconstruction [25, 33], but the graft type was not significantly associated with PF joint cartilage damage in this study. Van de Velde et al. [30] reported that ACL-reconstructed knees with a BPTB autograft induced a greater patellar lateral displacement and lateral tilt compared to the contralateral intact knees. Altered PF kinematics leads to an altered contact pressure of the PF cartilage; thus, a BPTB autograft is generally recognised as a risk factor for PF cartilage lesion. However, no significant correlation was found between BPTB autograft and new cartilage lesions in this study, and this finding is supported by several studies [17, 20].

Generally, PF OA is correlated with knee extension deficit [5]. Knee extension deficit early after ACL reconstruction or at the final follow-up was associated with OA changes [13, 24]. However, the present study revealed no significant correlation between preoperative and postoperative extension deficit and new PF cartilage lesion. Initial graft tension also affects PF joint biomechanics [9]. Therefore, initial graft tension has recently been implicated as the factor influencing PF cartilage [6]. A cadaveric study has revealed that the peak contact pressure at the PF joint is higher in ACL-reconstructed knees than in ACL-intact knees [10]. However, no significant difference in peak contact pressure was observed among the differing graft tensions. On the contrary, a recent study comparing the effects of difference initial graft tension on TF relationship revealed that higher initial graft tension leads to the external rotation of the tibia just after ACL reconstruction [27]. Furthermore, a previous cadaveric study revealed that the tibia moved posterolaterally with external rotation during the increase in the initial graft tension [15]. While the TF and PF joints were not distinguished, a recent study reported that a significant difference in OA outcome was found between the different initial graft tensions [3]. Therefore, the effect of initial graft tension on PF cartilage is of concern. However, the present study revealed no significant correlation between initial graft tension and new PF cartilage lesion.

Generally, the presence of cartilage damage during ACL reconstruction affects cartilage damage progression irrespective of surgery [1, 7, 32]. In particular, PF joint cartilage damage is aggravated after ACL reconstruction [8]. Notably, PF cartilage lesions at the primary surgery are linked to OA development in the PF joint at follow-up [4]. Similarly, TF cartilage lesions at primary surgery are a well-known risk factor for OA development in the PF joint [11]. Therefore, patients with ICRS grade of ≥ 1° PF compartment cartilage lesions or ≥ 2° TF compartment cartilage lesions during ACL reconstruction were excluded to distinguish between the progression of pre-existing and the occurrence of new cartilage lesions.

This study has several limitations. First, this retrospective study had limitations inherent to all retrospective studies. Second, this study included patients who agreed to second-look arthroscopy and hardware removal, which allows for selection bias. Third, we focused only on patients who underwent second-look arthroscopy 18 months postoperatively and had no PF and TF compartment cartilage lesions at primary ACL reconstruction; therefore, our sample size was limited. Fourth, cartilage was only evaluated by arthroscopy, and cartilage lesions were only evaluated using the ICRS articular cartilage injury classification. A combined analysis using MRI should be performed to accurately evaluate the quality of cartilage lesions. Fifth, a radiographic assessment for PF alignment was not conducted, although none of the patients had a history of patellar dislocation or patellar maltracking. Sixth, since the restricted protocol was employed for cases with repair of meniscus radial tear, the rehabilitation protocols were not identical and the rehabilitation protocol was only applied to three patients. Seventh, a control group of patients with ACL injury who did not undergo ACL reconstruction was not included. Moreover, including this control group was difficult as arthroscopy was used to assess cartilage lesion. Eighth, since ROM was not included as a variable in the multiple regression analysis, the interaction between ROM, especially extension deficit and quadriceps muscle strength, cannot be fully denied. Finally, the relation of cause and effect between quadriceps strength weakness and PF cartilage lesion is unclear because PF pain leads to quadriceps strength weakness [22]. Quadriceps strength at 1 year postoperatively may prevent PF articular cartilage lesions through postoperative rehabilitation, although evaluating the possibility of an interaction and concluding whether quadriceps strength weakness is a cause or an effect are difficult owing to the retrospective nature of this study and preoperative quadriceps strength rendered no effects.

## Conclusion

Quadriceps strength weakness 1 year after ACL reconstruction and the male sex were correlated with new PF cartilage lesion after anatomical ACL reconstruction. No significant correlation was found between BPTB autograft, initial graft tension, or extension deficit and new PF cartilage lesion. The clinical relevance of this study is as follows: rehabilitation that focuses on quadriceps strength after ACL reconstruction is recommended to prevent new PF cartilage lesions, particularly for male patients.

## Data Availability

The datasets used or analysed during the current study are available from the corresponding author on reasonable request.
